# Physicochemical, Antioxidant, Microbial, and Sensory Analysis of Buffalo Milk Stirred Yogurt Fortified With Cactus Pear ( *Opuntia ficus indica* ) Pulp

**DOI:** 10.1002/fsn3.70846

**Published:** 2025-08-27

**Authors:** Farzana Siddique, Muhammad Arshad, Rashid Ahmad Khan, Muhammad Qasim Ali, Sidrah, Muhammad Siddique Raza, Muhammad Naeem Zubairi, Nida Firdous, Ashiq Hussain, Henock Woldemichael Woldemariam

**Affiliations:** ^1^ Institute of Food Science and Nutrition University of Sargodha Sargodha Pakistan; ^2^ Department of Zoology University of Sargodha Sargodha Pakistan; ^3^ Faculty of Chemical and Process Engineering Technology Universiti Malaysia Pahang Kuantan Pahang Malaysia; ^4^ National Institute of Food Science and Technology University of Agriculture Faisalabad Pakistan; ^5^ Faculty of Food Science and Nutrition Bahauddin Zakariya University Multan Pakistan; ^6^ Department of Food Science and Technology MNS‐University of Agriculture Multan Multan Pakistan; ^7^ Department of Chemical Engineering, College of Engineering Addis Ababa Science and Technology University (AASTU) Addis Ababa Ethiopia

**Keywords:** antioxidant activity, cactus pear, flavored yogurt, syneresis susceptibility, total phenolic content, water holding capacity

## Abstract

The current study aimed to evaluate the impact of incorporating cactus pear pulp (CPP) at various concentrations (2%, 4%, 6%, 8%, and 10% w/w) levels on the physicochemical, functional, LAB, and sensory attributes of stirred buffalo milk yogurt during refrigerated storage over 21 days. The results revealed that all measured qualitative attributes of yogurt samples were significantly (*p* < 0.05) affected by the CPP concentrations and storage durations. The study showed that adding CPP to yogurt led to an increase in pH and a decreasing trend in acidity. The CPP yogurt samples (10% CPP) exhibited the highest pH (4.97) on Day 1. Significantly high (*p* < 0.05) titratable acidity was recorded in the control sample on Day 21 during storage. Syneresis susceptibility was reduced in 10% CPP added yogurt samples and demonstrated the lowest values (30.36) on 1st day of storage. Significantly (*p* < 0.05) enhanced water‐holding capacity (68.59%) in the yogurt supplemented with 10% CPP was observed. Increasing the amount of CPP in yogurt resulted in a constant increase in water‐holding capacity, while simultaneously lowering syneresis across the storage period. Functional parameters including total phenolic content and antioxidant activity showed a significant dose‐dependent increase and decreasing trend as a function of storage period. On the first storage day, yogurt samples having 10% CPP had the highest total phenolic content (8.22 mg GAE/100 g) and DPPH scavenging activity (83.45%). The viable count of LAB showed a substantial rise in CPP added yogurt samples on 1st day of storage, while a gradual decline was noted over the storage period in all treatments. Significant differences in sensory properties were observed among the samples; however, yogurt containing 4% CPP emerged as the most acceptable formulation based on sensory profiling. The findings concluded that adding CPP considerably enhanced the physicochemical, functional, LAB, and sensory attributes of yogurt samples.

## Graphical abstract

Cactus par pulp due to presence of bioactive compounds and fibers have huge potential to be incorporated in the stirred yogurt. Up to 10% incorporation of cactus pear pulp in buffalo milk stirred yogurt resulted significant increase in total phenolic content, antioxidant potential, and lactic acid bacteria count of the yogurt. Moreover, a significant decrease in the syneresis was observed. While, the yogurt formulation containing 4% cactus pear pulp emerged as the most acceptable formulation based on sensory profiling.

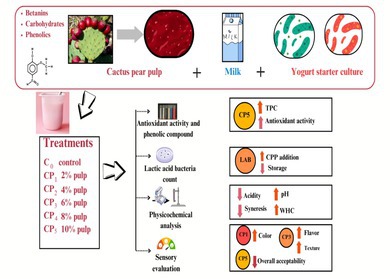

## 1 Introduction

Food products that are as fresh as possible, free of artificial additives and preservatives, and have excellent nutritional and sensory qualities have been at the forefront of consumer preferences and needs. Consumer demand for foods high in bioactive compounds, such as fiber, vitamins, pigments, minerals, terpenoids, and phenolic compounds, has increased due to the rise in chronic degenerative diseases like cancer, diabetes, obesity, and chronic heart failure (Hussain et al. 2022, 2023; Kalkan et al. 2025). Regular intake of naturally occurring and formulated plant‐based foods is required for an adequate intake of these compounds. Fruits and vegetables are excellent sources of bioactive compounds (Hussain et al. 2024, 2025). Cactus pear (*Opuntia ficus indica*) fruit is a perennial member of the Cactaceae family of cacti that are native to Mexico and thrive in semi‐arid and dry environments (Trujillo et al. 2023). Mexico accounts for over half of global cactus pear cultivation. After Mexico and Italy (12.2%), South Africa (3.7%) comes in with the next highest percentages. Cactus pear cultivars have a vast variety of skin and flesh colors. This variety can go from green to white, yellow to orange, or red to purple (De Wit et al. 2020; Abbas et al. 2022). These color differences are especially noticeable in the plants growing in their natural and wild habitats. The entire cactus pear fruit can be divided into two parts: the non‐edible part, which consists of the peel and seeds and is typically thrown away, and the edible pulp, which can be eaten raw or processed into juices, nectars, jams, jellies, frozen syrups, and pulp (Manzur‐Valdespino et al. 2022; Van Rooyen et al. 2024). For centuries, fresh cladodes, fruits, and flowers from the cactus pear have been used for a variety of reasons in customs that date back centuries. Cactus pear fruits have great nutritional characteristics and are commercially valuable. They are an excellent source of biologically active compounds like polysaccharides, proteins, polyphenols, vitamins, minerals, and flavonoids (Kiranmai 2022; Ammar et al. 2018; Muñoz‐Tebar et al. 2025). These compounds are linked with numerous achievable health advantages, including anti‐inflammatory, immune‐modulatory, antidiabetic, analgesic, and antioxidant qualities. Phenolic substances with antioxidant characteristics present in cactus pear have been identified as important contributions to human health protection against a variety of ailments, including bronchial irritation, acid reflux, hyperglycemia, elevated blood cholesterol levels, arteriosclerosis, and cardiovascular and stomach ailments (De Wit et al. 2019; Baspinar and Güldaş 2021; Cifelli et al. 2020). Because of these qualities, the CPP could be considered as perfect for the food sector as a functional ingredient with extra health advantages as well as a food component.

Yogurt is a traditional sour dairy food made by fermentation of milk from various mammals, by the action of *Lactobacillus bulgaricus* and *Streptococcus thermophilus* . Yogurt is packed with essential nutrients, offering potassium, calcium, protein, and B group vitamins for a well‐rounded health boost (Vatanparast et al. 2019; Kalkan et al. 2025). Yogurt consumption has significantly risen all over the world thanks to its high nutritional profile along with functional and therapeutic characteristics (Gómez‐Gallego et al. 2018; Kok and Hutkins 2018). The selection of milk for the development of yogurt is an important indicator of the desired results in the final products. Because buffalo milk is higher in fat, protein, and specific vitamins and minerals, it is regarded as superior to milk from cows, sheep, and goats. Additionally, it has a deeper, creamier flavor and works well to make dairy goods (Emakpor et al. 2024; Rashid et al. 2024). As a result, in the current work, buffalo milk was chosen to create stirred yogurt with CPP addition at various levels. Yogurt recipes can be enhanced with fruit in the form of blends, syrups, and juices (Salehi et al. 2021; Sharifi et al. 2023). The FAO and WHO suggest a 5%–15% fruit concentration while preparing yogurt. That is why the market for fruit‐flavored yogurts is growing. Cactus pear fruit is a cost‐effective source that can be utilized in the development of innovative food products (Andreu‐Coll et al. 2020). Muñoz‐Tebar et al. (2025), for instance, evaluated the impact of adding flours made from cactus pear peel and pulp on the physicochemical parameters, microbiological, antioxidant capacity, and sensory qualities of sheep milk yogurts. Nevertheless, they only added 1% of cactus pear powder to the yogurt recipes. Similarly, Albayati et al. (2024) also used CPP to develop set yogurts from cow milk; however, their analysis included storage studies for only 2 weeks. Taheur et al. (2023) revealed that the addition of cactus pear fruit powder with kefir produced a dairy‐fermented product with higher antioxidant and phenolic content. Additionally, the syneresis was decreased, and the majority of sensory qualities were enhanced. However, the stability and antioxidant activity of bioactive compounds from CPP during yogurt production and storage were unexplored in that study. Therefore, this work addresses the need to investigate the value‐adding of CPP for the development of stirred yogurt from buffalo milk, given the growing consumer demand for foods with improved nutritional profiles and health benefits, as well as the potential of CPP as sources of functional ingredients and the suitability of yogurt as a vehicle for them. Thus, in the present research, yogurt was developed by the addition of CPP at relatively higher levels (up to 10%). The research aimed to enhance the nutritional and antioxidant properties of stirred yogurt. The research examined how the addition of CPP impacts the pH, titratable acidity, syneresis susceptibility, water holding capacity, total phenolic content, DPPH scavenging activity, total LAB viable count, and sensory attributes of the yogurt.

## 2 Materials and Methods

### 2.1 Sample Procurement and Preparation

Cactus pear was collected from the city of Talagang, Pakistan. Cactus pear fruits were gently washed with tap water, peeled aseptically with a stainless steel knife, and subjected to extraction of pulp. The seeds were removed from the extracted pulp by passing it through a sieve. The fine pulp was then pasteurized, filled in glass jars, and refrigerated stored at 4°C for addition in the yogurt. All reagents used in the study were of analytical grade. Gallic acid, Folin–Ciocalteu, and 2‐diphenyl‐1‐picrylhydrazyl (DPPH) were supplied by Sigma‐Aldrich (Sigma‐Aldrich, Darmstadt, Germany).

### 2.2 Proximate Analysis of Cactus Pear Pulp (CPP)

Proximate analysis of cactus pear pulp (CPP) was conducted using the methods described in the AOAC. These were examined using the gravimetric method (AOAC method No. 934.01), dry incineration in a muffle furnace (AOAC method No. 976.05), and Soxhlet method (AOAC method No. 954.02) for moisture, ash, and crude fiber, respectively. Protein content was measured using the Kjeldahl method following AOAC method 976.05. Nitrogen content obtained through this analysis was then converted to total protein concentration by applying a conversion factor of 6.25. The total carbohydrate content was calculated using the difference approach, as shown in the below equation:

$$\text{Total carbohydrate}\;\left(\%\right)=\left[100-\left(\text{Moisture}+\text{Protein}+\mathrm{Fat}+\mathrm{Ash}\right)\right]$$

### 2.3 Phytochemical Analysis of Cactus Pear Pulp (CPP)

The total phenolic content (TPC), total flavonoid content (TFC) and DPPH free radical scavenging activity of CPP through hydroethanolic extraction (70; 30 v/v) were determined using spectrophotometric methods at 765, 550, and 517 nm, respectively. These methods have been described in detail by Hussain et al. (2021).

### 2.4 Yogurt Formulation With Cactus Pear Pulp (CPP) Addition

Buffalo milk (4.46% protein, 7.2% fat, 0.81% ash, and 16.73% total solids) was pasteurized at 90°C for 10 min. Then 2% Direct Vat Set (DVS) culture (ThermophilicYoFlex starting) containing *Lactobacillus bulgaricus* and *Streptococcus thermophilus* (Chr. Hansen Horsholm, Denmark) was added after cooling (42°C ± 1°C) and left for incubation at 42°C–44°C. Once 4.6 pH was reached, the samples of yogurt were ready. To halt acidification, the yogurt samples were cooled to 4°C immediately. After cooling, CPP (pasteurized and cooled previously) was added to the yogurt at various concentrations [2%, 4%, 6%, 8%, and 10% (w/w)] and mixed thoroughly to ensure uniform dispersion in stirred‐type yogurt. The formulated yogurt samples containing CPP were then stored at refrigeration temperature (4°C) for further analysis. In the results section, all initial pH readings correspond to Day 1 of storage, recorded after CPP was added. The measured values thus reflect the pH of the final formulated product, not the base yogurt post‐fermentation. All the stirred CPP yogurt samples were kept at a refrigeration temperature (4°C ± 1°C) for 21 days of storage. All of the experiments were conducted three times to ensure result reliability (*n* = 3).

### 2.5 Determination of DPPH Free Radical Scavenging Activity

The antioxidant capacity of yogurt treatments containing CPP was assessed using the DPPH assay, according to the methodology provided by Hussain et al. (2021). Briefly, 1 g of yogurt was homogenized with ten milliliters of ethanol. After combining 0.2 milliliters of the specified solution with 0.8 milliliters of DPPH solution at a concentration of 1.5 × 10^−4^ M, the mixture was allowed to stand for 30 min in an area devoid of light. Absorbance readings were obtained utilizing a UV spectrophotometer (UV‐1800; Shimadzu Instruments Mfg. Co. Ltd.) at a wavelength of 517 nm. All of the measurements were done three times. The following equation was used to calculate the degree of discoloration: The DPPH scavenging activity was calculated using the following equation;

$$\text{Inhibition}\;\left(\%\right)={\mathrm{Abs}}_{\left(\text{control}\right)}-{\mathrm{Abs}}_{\left(\text{sample}\right)}/{\mathrm{Abs}}_{\left(\text{control}\right)}\times 100$$

Abs sample denotes the absorbance of DPPH radical with yogurt samples, and Abs control represents the absorbance of DPPH radical with ethanol.

### 2.6 Assessment of Total Phenolic Content (TPC)

Total phenolic content (TPC) was calculated according to the Folin–Ciocalteu method as mentioned by (Hussain et al. 2021), with required modifications. Briefly, 100 μL of yogurt extract, 7.9 mL of distilled water, and 0.5 mL of Folin–Ciocalteu reagent (2 M) were combined in a test tube. Samples were allowed to stand in the dark at room temperature for a few minutes before being subjected to a solution of sodium carbonate (1.5 mL of 20% w/v). The spectrophotometer's absorbance was measured at a wavelength of 765 nm.

TPC in gallic acid equivalents (μg GAE/mL) was determined based on the equation below:

$$\mathrm{TPC}\;\left(\mathrm{\mu g}\;\mathrm{GAE}/\mathrm{mL}\right)=C\times V/M$$

*C*, *V*, and *M* stand for the sample's mass, volume, and concentration of gallic acid, respectively, as determined by the calibration curve.

### 2.7 Physicochemical Analysis

#### 2.7.1 pH and Titratable Acidity

The pH value of the samples was determined following the AOAC method 943.02 (AOAC 2000) using a pH meter (pH 211 Microprocessor pH meter; Hanna Instruments). Yogurt samples were homogenized in water at a ratio of 1:9 before pH assessment. Titratable acidity was measured by titrating the samples with 0.1 N sodium hydroxide (NaOH) solution until a pink tint appeared and was expressed as a lactic acid percentage.

#### 2.7.2 Total Solids

Total solids were measured by following the (AOAC 2000) method. For this purpose, 2 g of yogurt were stored in a desiccator for 30 min after being heated to 100°C for 3 h. Total solids represented the proportion of residues that were obtained.

$$\begin{array}{c} \text{Total Solids}\;\left(\%\right)\\ =\text{Weight of sample after drying}/\text{Weight of sample before drying}\times 100 \end{array}$$

#### 2.7.3 Water Holding Capacity (WHC)

WHC was calculated by following the procedure outlined by (Ferweez et al. 2023). Briefly, 5 g of each sample was centrifuged at 5°C and 4500 × *g* for 15 min. The whey was collected and weighed after centrifugation. WHC was computed using the subsequent formula:

$$\mathrm{WHC}\;\left(\%\right)=\left[1-\left(W1/\text{W2}\right)\right]\times 100$$

W1 is the weight of drained whey, and W2 is the sample's original weight.

#### 2.7.4 Syneresis

The syneresis was measured using the method given by Dong et al. (2022). Each yogurt sample weighed 200 g and was centrifuged at 770 × *g* for 10 min at 4°C to calculate the syneresis value.

$$\begin{array}{c} \text{Syneresis}\;\left(\%\right)\\ =\text{Total}\;\mathrm{wt}.\;\text{of separated liquid}\;\left(g\right)/\text{Total}\;\mathrm{wt}.\;\text{of yogurt sample}\;\left(g\right)\times 1 \end{array}$$

### 2.8 Determination of Lactic Acid Bacteria (LAB) Viable Count

The LAB viable count was obtained using the method given by (Afiyah et al. 2023). The yogurt samples undergo serial dilution using peptone water. The MRS and M17 agar were used for the enumeration of *Lactobacillus bulgaricus* and *Streptococcus thermophilus* , respectively. This procedure followed the pour‐plate method. To count *Lactobacillus bulgaricus* and *Streptococcus thermophilus*, the plates were incubated for 72 h at 37°C and 48 h at 42°C, respectively. The findings were expressed as log of colony‐forming units per milliliter (log CFU/mL).

### 2.9 Sensory Evaluation

The sensory qualities of yogurt were examined using the approach proposed by Rafique et al. (2023). The sensory characteristics of the yogurt samples, such as color, flavor, and texture, were assessed by the trained panelists in the laboratory using a 9‐point hedonic scale. The yogurt formulations were evaluated by semi‐expert panel of 60 people, males and females, having ages between 20 and 55 years. For the evaluation of yogurt, 30 mL samples that had been refrigerated (5°C) were presented in 100 mL transparent plates and covered with plastic lids. Evaluations were carried out in white light and at room temperature (25°C). Informed consent was obtained from the evaluators, and they were allowed to withdraw at any stage of evaluation.

### 2.10 Statistical Analysis

All experiments were conducted in distinct batches, and triplicate measurements were recorded for each. The experimental results were presented as mean values along with their corresponding standard deviations. The data was statistically analyzed using ANOVA, employing a significance threshold of *p* < 0.05, and means were compared by Tukey's post hoc test. The data from the gathered values was analyzed using software called SPSS Statistics 8.0 from IBM in the USA (Turgut and Diler 2023).

## 3 Results and Discussion

### 3.1 Chemical and Functional Composition of Cactus Pear Pulp (CPP)

Table 1 outlines the chemical and functional composition of CPP on a dry weight basis, including moisture, protein, fat, crude fiber, ash, and carbohydrate content in grams per 100 g. For CPP, the moisture content was found to be 86.55%, protein content 0.83%, fat content 0.24%, crude fiber 1.13%, ash 0.26%, and carbohydrate 10.29%. Additionally, in the CPP, the total phenolic content was found to be 218.8 mg GAE/100 g, antioxidant activity 57.12%, and total flavonoid content 112.75 mg QE/100 g. The results regarding the proximate composition of CPP have also been confirmed by the findings of Al‐Mushhin (2022), who also reported that CPP is high in moisture and carbohydrates and low in fat, while having a notable amount of ash, fiber, and protein. Current findings have also been confirmed by Albergamo et al. (2022), as they reported protein content 0.78%, fat 1.12%, ash 0.28%, fiber 0.46%, moisture 16.57%, and carbohydrates 74.34%. The results regarding phenolics and flavonoids and antioxidant activity of CPP are in line with the findings of De Wit et al. (2019), when they investigated different cultivars of cactus pear and observed that all are a good source of phenolics and flavonoids. They also observed the strong antioxidant potential of CPP. Findings of Lugo‐Zarate et al. (2021) have also confirmed that CPP is a good source of phenolics and flavonoids, and due to the presence of these bioactive compounds, CPP has the potential to exhibit antioxidant activity. The current findings about CPP are higher than those of Lamia et al. (2018), who found that *Opuntia streptacantha* had a TPC of roughly 104.66 ± 1.52 mg GAE/100 g DW, while the methanolic pulp extract of *Opuntia ficus indica* contained roughly 54.33 ± 2.51 mg GAE/100 g. In a similar vein, the TFC values for CPP are also higher than those reported by Lamia et al. (2018), who found that the TFC in pulp from *Opuntia* was 22.47 ± 2.1 mg RE/100 g DW. This could be because the authors used a different solvent in the current investigation, which produced hydroethanolic extracts with significant phenolic and flavonoid concentrations. However, variations in the bioactive compounds are also caused by variations in the cultivar and regional circumstances (Iftikhar et al. 2023). Present findings have also been supported by the results provided by Al‐Mushhin (2022), as they also reported appreciable amounts of TPC, TFC, and antioxidant activity presented by CPP.

**TABLE 1:** Chemical and functional composition of cactus pear pulp.

Parameters	Quantity
Moisture (%)	86.55 ± 0.35
Protein (%)	0.83 ± 0.14
Fat (%)	0.24 ± 0.17
Crude fiber (%)	1.13 ± 0.08
Ash (%)	0.26 ± 0.11
Carbohydrates (%)	10.29 ± 0.39
Total phenolic content (mg GAE/100 g)	218.8
Total flavonoid content (mg QE/100 g)	112.75
Antioxidant activity (%)	57.12

Abbreviations: GAE, gallic acid equivalent; QE, querecetin equivalent.

### 3.2 pH and Titratable Acidity of Yogurt

Table 2 illustrates the outcomes of CPP‐supplemented and controlled yogurt samples during a storage period of 1–21 days. The statistical analysis showed that the pH value of yogurt samples significantly varied by the change in CPP concentration and storage period. Among various yogurt samples, the overall highest pH value (4.79 ± 0.03) was observed in the yogurt sample containing 10% fruit pulp, whereas the overall lowest pH value (4.48 ± 0.02) was observed in the control sample. Similarly, during storage, the overall highest pH value (4.75 ± 0.02) was observed in the yogurt sample on the 1st day of storage, whereas the lowest pH value (4.53 ± 0.03) was observed in the yogurt sample on the 21st day of storage.

**TABLE 2:** pH and titratable acidity of yogurt.

Parameter	Sample code	Storage (days)				
		1	7	14	21	Overall, means ± SD
pH	C	4.60 ± 0.01^j^	4.51 ± 0.03^l^	4.37 ± 0.02^o^	4.43 ± 0.02^n^	4.48 ± 0.02^D^
	CP_1_	4.62 ± 0.00^i^	4.58 ± 0.04^jk^	4.52 ± 0.03^l^	4.47 ± 0.01^m^	4.55 ± 0.02^CD^
	CP_2_	4.65 ± 0.03^h^	4.55 ± 0.04^k^	4.48 ± 0.01^m^	4.45 ± 0.02^n^	4.63 ± 0.03^bc^
	CP_3_	4.78 ± 0.02^d^	4.72 ± 0.00^e^	4.57 ± 0.02^k^	4.52 ± 0.01^l^	4.65 ± 0.01^B^
	CP_4_	4.86 ± 0.05^b^	4.74 ± 0.02^e^	4.61 ± 0.03^ij^	4.64 ± 0.04^h^	4.71 ± 0.04^AB^
	CP_5_	4.97 ± 0.03^a^	4.81 ± 0.04^c^	4.69 ± 0.01^f^	4.67 ± 0.05^g^	4.79 ± 0.03^A^
	Overall, means ± SD	4.75 ± 0.02^A^	4.65 ± 0.03^B^	4.54 ± 0.02^C^	4.53 ± 0.03^C^	
Titratable acidity (%)	C	0.97 ± 0.02^d^	0.99 ± 0.04^c^	1.07 ± 0.02^b^	1.06 ± 0.03^b^	1.02 ± 0.03^A^
	CP_1_	0.96 ± 0.03^d^	0.95 ± 0.05^e^	0.99 ± 0.04^c^	1.13 ± 0.01^a^	1.01 ± 0.03^A^
	CP_2_	0.92 ± 0.02^f^	0.96 ± 0.01^d^	0.95 ± 0.05^e^	0.99 ± 0.01^c^	0.95 ± 0.02^B^
	CP_3_	0.88 ± 0.03^h^	0.89 ± 0.02^g^	0.90 ± 0.01^g^	0.95 ± 0.02^e^	0.90 ± 0.02^C^
	CP_4_	0.83 ± 0.05^i^	0.87 ± 0.02^h^	0.89 ± 0.01^g^	0.90 ± 0.04^g^	0.87 ± 0.03^C^
	CP_5_	0.78 ± 0.01^j^	0.78 ± 0.04^j^	0.77 ± 0.01^k^	0.79 ± 0.03^j^	0.78 ± 0.02^D^
	Overall, means ± SD	0.89 ± 0.03^B^	0.90 ± 0.03^B^	0.93 ± 0.02^AB^	0.97 ± 0.02^A^	

*Note:* Values are the mean ± standard deviation. Means carrying different superscript letters within a column indicate significant differences (*p* < 0.05) among CPP concentration. Letters within a row indicate significant differences (*p* < 0.05) within a sample over storage time.

Abbreviations: C, Control yogurt; CP_1_, Yogurt supplemented with 2% CPP; CP_2_, Yogurt supplemented with 4% CPP; CP_3_, Yogurt supplemented with 6% CPP; CP_4_, Yogurt supplemented with 8% CPP; CP_5_, Yogurt supplemented with 10% CPP.

Initially, the pH of control samples over storage duration remained within the range of 4.60 to 4.43. Yogurt samples showed declining tendencies in pH as the storage period increased from 7 to 21 days. As for CPP‐supplemented samples, the pH ranged from 4.62 to 4.47 for 2% CPP, 4.65 to 4.45 for 4% CPP, 4.78 to 4.52 for 6% CPP, 4.86 to 4.64 for 8% CPP, and 4.97 to 4.67 for 10% CPP during all storage durations. Notably, the control yogurt had the lowest pH (4.60 ± 0.01) on Day 1. While the supplemented yogurt samples showed a concentration‐dependent increasing trend during all storage durations. Similarly, on Days 7, 14, and 21, the pH ranges were observed to be 4.51–4.81, 4.37–4.69, and 4.43–4.67, respectively. Across all these days, as the levels of CPP addition increased from 2% to 10%, there was a slight increase in pH values in all fortified samples. The pH increase in 2% and 4% supplemented samples was not significant (*p* > 0.05) compared to control. However, 8% and 10% fortified samples had significantly higher (*p* < 0.05) pH values on Days 14 and 21, compared to the control. The inclusion of CPP might have neutralized the lactic acid in the yogurt samples, potentially contributing to the observed trend of increasing pH values in the supplemented samples during storage (Basiony et al. 2023). Furthermore, CPP has inherently higher pH (~6.1), and its incorporation might elevate the pH of the CPP yogurt formulations; these results are in accordance with previous researchers (Amal et al. 2016; Ahmed et al. 2022). The observed increasing trend in pH is due to slow acidification, indicating a potential disruption of the symbiotic relationship among initial bacterial strains due to whey separation induced by the addition of fruit pulp (Jung et al. 2016; Minj et al. 2019).

Turning to titratable acidity, the mean values indicated that the titratable acidity of yogurt samples significantly (*p* < 0.05) varied by the CPP concentration levels and storage days. Overall, the highest titratable acidity (1.02 ± 0.03) was observed in control yogurt, whereas the lowest (0.78 ± 0.02) was observed in yogurt samples containing 10% CPP. The titratable acidity ranged from 0.92% to 0.99%. Similarly, during storage, the overall highest titratable acidity (0.97 ± 0.02) was observed in yogurt samples on the 21st day of the storage period, whereas the lowest (0.89 ± 0.03) was observed on 1st day of the storage period. Table 2 shows that 10% CPP supplementation samples maintained a relatively consistent titratable acidity between 0.78% and 0.79% across all storage periods. Titratable acidity in 8% CPP‐supplemented samples stored for 1–21 days ranged from 0.83% to 0.90%. Meanwhile, 6% CPP‐supplemented yogurt samples had a titratable acidity of 0.88%–0.95% across all storage times. For yogurt samples added with 4% CPP, the titratable acidity ranged from 0.92% to 0.99%. The titratable acidity for samples containing 2% CPP supplementation was 0.96%–1.06%, and control samples showed 0.97%–1.13% acidity during storage. Overall, titratable acidity decreased proportionally with CPP concentration, showing inverse relation with storage period. Similar results were observed by Elizabeth and Onyinyechi (2012), who reported that the titratable acidity of yogurt samples decreased with the addition of beetroot juice. These trends are also related to the findings of Mahmood et al. (2008) and Tarakci (2010), who reported that the addition of additives with low acidity can reduce the acidity of yogurt to which they are added. Earlier researchers reported that the incorporation of some fruit powders/flours into yogurt samples may lead to a decreasing trend in total acidity, slowing down the acidification phenomenon. Previous study results of Singh et al. (2018) are also in agreement with the present study, which reported that the buffering capacity of certain fruit fibers or phenolic compounds resulted in lower post‐fermentation acidity as observed with the addition of pineapple peel powder in yogurt samples. Similarly, Ahmed et al. (2021) demonstrated that fortifying yogurt with fig extract led to a higher pH and lower titratable acidity compared to control, further confirming the present findings. Current findings are further supported by Hashemi et al. (2019), who demonstrated that yogurt enriched with plant‐based additives showed reduced acidity during storage. These findings clearly suggest that the acidity trend observed in yogurt samples might be dependent on the inherent biochemical composition as well as the microbial compatibility of the added ingredient. Notably, this modulation can be advantageous in creating yogurt with balanced acidity, improved sensory profile, and enhanced functional properties, provided that the fermentation parameters are optimized for each fruit type.

### 3.3 Syneresis Susceptibility and Water Holding Capacity (WHC) of Yogurt

WHC and syneresis were assessed in all CPP‐fortified (2%, 4%, 6%, 8%, and 10%) and control samples of yogurt. Table 3 summarizes the results regarding syneresis susceptibility, showing a significant (*p* < 0.05) decrease in syneresis susceptibility with increasing levels (2% to 10%) of CPP. The control sample showed the highest average syneresis (36.84% ± 1.22%), while the lowest value (30.62% ± 1.44%) was recorded in CP5. CPP‐fortified samples exhibited consistently lower syneresis values during all storage intervals (1, 7, 14, and 21 days), compared to the control, with minimal statistical variations over time, suggesting that storage duration had a non‐significant effect on the syneresis within treatments. Contrary to syneresis susceptibility, WHC significantly (*p* < 0.05) increased with CPP concentration levels from 2% to 10%. The control sample indicated the lowest WHC value (55.65% ± 1.60%), whereas CP5 (10% CPP) exhibited the highest value (67.70% ± 1.62%). This increasing trend in WHC value is directly linked to the water entrapment properties of the CPP. The increasing trend of WHC and decreasing syneresis susceptibility was consistent and complementary, suggesting a strong negative correlation between syneresis and WHC. The results indicated that enriched yogurt samples had decreased syneresis tendencies, with increases in CPP levels from 2% to 10%. Existing literature suggests that dietary fiber sourced from plants may exhibit an absorptive effect, influencing the retention of serum or the release of whey from the gel matrix of yogurt (Raza et al. 2021; Arab et al. 2023). The rising WHC and declining trend in syneresis of yogurt samples might be associated with the inclusion of CPP, as reported by earlier researchers. Öztürk et al. (2018) suggested that this happening may be linked to the increased water binding ability of CPP. This addition may have increased the accessible carbs, fiber, and pectic compounds that enhance water retentive power (Ondarza 2016). The findings of the present study coordinate with the study of Barkallah et al. (2017), indicating that the inclusion of Spirulina in yogurt samples having higher dietary fiber content decreased the occurrence of syneresis in treatment groups, in contrast to the control group. Moreover, the consistent decrease in syneresis observed in supplemented yogurt samples was linked to reduced serum whey expulsion, consequently enhancing water retention within CPP supplemented yogurt samples, supported by studies of Kermiche et al. (2018) and Jridi et al. (2015), who confirm that improved WHC is inversely associated with syneresis, reinforcing the present findings.

**TABLE 3:** Syneresis susceptibility and water holding capacity of yogurt.

Parameter	Sample code	Storage (days)				
		1	7	14	21	Overall, means ± SD
Syneresis susceptibility (%)	C	36.85 ± 0.58^a^	36.02 ± 0.46^b^	37.51 ± 1.80^a^	36.98 ± 2.04^a^	36.84 ± 1.22^A^
	CP_1_	35.71 ± 1.24^b^	35.93 ± 2.16^b^	36.11 ± 0.66^b^	36.00 ± 1.52^b^	35.94 ± 1.40^B^
	CP_2_	34.53 ± 1.40^c^	34.84 ± 1.62^c^	35.07 ± 0.80^c^	34.89 ± 1.38^c^	34.83 ± 1.30^C^
	CP_3_	32.17 ± 2.26^f^	32.99 ± 0.54^e^	33.36 ± 1.46^e^	33.94 ± 1.10^d^	33.12 ± 1.34^D^
	CP_4_	30.28 ± 1.32^g^	30.73 ± 1.78^g^	31.26 ± 2.02^fg^	31.36 ± 1.28^fg^	30.91 ± 1.55^E^
	CP_5_	30.36 ± 1.36^g^	30.50 ± 2.10^g^	30.81 ± 1.52^g^	30.82 ± 1.24^g^	30.62 ± 1.44^E^
	Overall, means ± SD	33.32 ± 1.36^A^	33.50 ± 1.44^A^	34.02 ± 1.38^A^	33.99 ± 1.43^A^	
WHC (%)	C	56.30 ± 0.83^j^	56.07 ± 1.46^j^	55.35 ± 2.10^k^	54.88 ± 2.04^k^	55.65 ± 1.60^E^
	CP_1_	58.73 ± 1.58^h^	57.92 ± 1.84^h^	57.19 ± 2.23^i^	58.10 ± 1.36^h^	57.99 ± 1.75^D^
	CP_2_	61.84 ± 1.28^f^	61.72 ± 0.64^f^	61.00 ± 1.33^f^	60.31 ± 1.20^g^	61.22 ± 1.11^C^
	CP_3_	65.77 ± 1.69^c^	64.81 ± 1.66^d^	64.39 ± 0.92^e^	64.94 ± 1.54^d^	64.98 ± 1.45^B^
	CP_4_	66.28 ± 2.26^c^	65.12 ± 0.60^d^	65.02 ± 1.86^d^	64.38 ± 1.02^e^	65.20 ± 1.44^B^
	CP_5_	68.59 ± 1.87^a^	67.99 ± 1.75^a^	67.21 ± 1.90^b^	67.01 ± 0.98^b^	67.70 ± 1.62^A^
	Overall, means ± SD	62.92 ± 1.58^A^	62.27 ± 1.33^B^	61.69 ± 1.72^C^	61.60 ± 1.36^C^	

*Note:* Values are the mean ± standard deviation. Means carrying different superscript letters within a column indicate significant differences (*p* < 0.05) among CPP concentration. Letters within a row indicate significant differences (*p* < 0.05) within a sample over storage time.

Abbreviations: C, Control yogurt; CP_1_, Yogurt supplemented with 2% CPP pulp; CP_2_, Yogurt supplemented with 4% CPP pulp; CP_3_, Yogurt supplemented with 6% CPP pulp; CP_4_, Yogurt supplemented with 8% CPP pulp; CP_5_, Yogurt supplemented with 10% CPP pulp.

### 3.4 Total Phenolic Content and DPPH Scavenging Activity

Polyphenols are essential for the color and flavor of food, despite the fact that they can be volatile and oxidation‐prone. Their antioxidant qualities aid in preserving color stability and averting unfavorable oxidation‐induced alterations (Cao et al. 2021). The total phenolic content (TPC) of yogurt samples was significantly (*p* < 0.05) influenced by both the concentration of CPP and storage duration (Table 4). The results revealed that the control yogurt had the lowest TPC (0.50 μg GAE/mL), while the yogurt containing 10% CPP had the highest TPC (8.22 μg GAE/mL). Although natural phenolics may degrade in the chemical reactions during storage, resulting in decreased TPC with time, this gradual reduction in phenolic content over storage time ought to be due to oxidative degradation, polymerization, or interactions with milk proteins, as also reported by earlier researchers (Barkallah et al. 2017). The increase in TPC with CPP addition in yogurt samples can be attributed to the betacyanins, flavonoids, and phenolic acids, like bioactive compounds reported in CPP. These compounds are known to possess strong antioxidant properties. Similar increases in TPC were reported by Ahmed et al. (2022) in yogurt fortified with pomegranate peel and by Amal et al. (2016) when CPP and papaya pulp were added. This suggests that CPP can serve as a valuable natural source of phenolics for functional dairy product development.

**TABLE 4:** Total phenolic content and DPPH scavenging activity of yogurt.

Parameter	Sample code	Storage (days)				
		1	7	14	21	Overall, means ± SD
TPC (μg GAE/mL)	C	0.57 ± 0.01^s^	0.54 ± 0.01^s^	0.53 ± 0.03^s^	0.50 ± 0.02^s^	0.54 ± 0.02^F^
	CP_1_	3.61 ± 0.01^p^	3.48 ± 0.04^q^	3.37 ± 0.01^q^	3.25 ± 0.02^r^	3.43 ± 0.02^E^
	CP_2_	4.86 ± 0.05^l^	4.58 ± 0.03^m^	4.42 ± 0.01^n^	4.19 ± 0.01^o^	4.51 ± 0.03^D^
	CP_3_	5.35 ± 0.02^i^	5.23 ± 0.01^j^	5.11 ± 0.02^k^	5.02 ± 0.05^k^	5.18 ± 0.03^C^
	CP_4_	7.18 ± 0.01^e^	7.03 ± 0.01^f^	6.87 ± 0.01^g^	6.60 ± 0.02^h^	6.92 ± 0.01^B^
	CP_5_	8.22 ± 0.04^a^	8.10 ± 0.01^b^	7.98 ± 0.03^c^	7.74 ± 0.02^d^	8.01 ± 0.03^A^
	Overall, means ± SD	4.97 ± 0.02^A^	4.83 ± 0.02^B^	4.71 ± 0.02^C^	4.55 ± 0.02^D^	
DPPH activity (%)	C	6.49 ± 0.21^m^	4.73 ± 0.26^o^	5.20 ± 0.46^n^	5.07 ± 0.18^n^	5.37 ± 0.28^F^
	CP_1_	62.50 ± 0.40^i^	60.96 ± 0.62^j^	54.27 ± 0.98^l^	57.66 ± 0.25^k^	58.85 ± 0.56^E^
	CP_2_	69.19 ± 1.04^f^	68.01 ± 0.48^g^	64.79 ± 0.26^h^	61.98 ± 0.52^i^	65.99 ± 0.58^D^
	CP_3_	72.71 ± 0.36^e^	71.44 ± 0.51^f^	68.13 ± 0.40^g^	68.52 ± 0.23^g^	70.20 ± 0.38^C^
	CP_4_	79.42 ± 1.28^b^	73.97 ± 0.41^e^	75.70 ± 0.19^d^	73.55 ± 0.66^e^	75.66 ± 0.64^B^
	CP_5_	83.45 ± 0.94^a^	80.28 ± 0.62^b^	79.91 ± 0.75^b^	77.86 ± 0.30^c^	80.38 ± 0.65^A^
	Overall means ± SD	62.29 ± 0.71^A^	59.90 ± 0.48^B^	58.00 ± 0.50^C^	57.44 ± 0.36^C^	

*Note:* Values are the mean ± standard deviation. Means carrying different superscript letters within a column indicate significant differences (*p* < 0.05) among CPP concentration. Letters within a row indicate significant differences (*p* < 0.05) within a sample over storage time.

Abbreviations: C, Control yogurt; CP_1_, Yogurt supplemented with 2% CPP pulp; CP_2_, Yogurt supplemented with 4% CPP pulp; CP_3_, Yogurt supplemented with 6% CPP pulp; CP_4_, Yogurt supplemented with 8% CPP pulp; CP_5_, Yogurt supplemented with 10% CPP pulp.

Table 4 also shows the influence of CPP concentration and storage period on yogurt's antioxidant activity (DPPH assay). The antioxidant potential of yogurt samples, measured as DPPH free radical scavenging activity, also showed significant (*p* < 0.05) improvement with increasing CPP levels. The antioxidant activity of control samples varied from 6.49% to 5.07% across all storage periods. The antioxidant activity of samples treated with various CPP concentration (2%–10%) ranged from a minimum value of 62.50% to a maximum value of 83.45% in 10% CPP‐supplemented yogurt samples during storage. Overall outcomes indicated that all CPP‐supplemented samples exhibited increased antioxidant activity in a concentration‐dependent manner throughout the storage duration. On the 1st day, the yogurt containing 10% CPP exhibited the highest antioxidant potential, with a recorded activity of 83.45% compared to the control with 6.49% (Table 4). The inclusion of CPP may increase antioxidant potential because of the abundance of phytochemicals found in CPP. The outcomes of this study may be related to the findings of Ahmed et al. (2022), who added pomegranate peels to yogurt and investigated their impact on the product's antioxidant activity. According to Amal et al. (2016) adding CPP and papaya pulp to yogurt increases its DPPH free radical scavenging activity. Adding both pineapple peel and pomace to yogurt had a similar effect on improving the antioxidant properties of the yogurt, as shown by the comparable results obtained from Sah et al. (2016). The increased antioxidant activity noticed in soured milk goods is attributed to the liberation of bioactive peptides through the proteolytic action of lactic acid bacteria, as noted by Guha et al. (2021). These findings were consistent with those of Marand et al. (2020), whose study revealed that the tyrosine amino acid in the fortified yogurt contributes to increases in antioxidant activity. Tyrosine possesses a phenolic side chain in its structure, attributing enhanced antioxidant properties to fortified yogurt (Del Galdo et al. 2018). Furthermore, the elevated antioxidant capacity of the enriched yogurt samples might be related to the presence of a higher concentration of anthocyanins. Anthocyanins are a collection of chemicals that contribute to the coloration of fruits, plants, and flowers (Anuyahong et al. 2020).

### 3.5 Lactic Acid Bacteria (LAB) Viable Count of Yogurt

**FIGURE 1:**
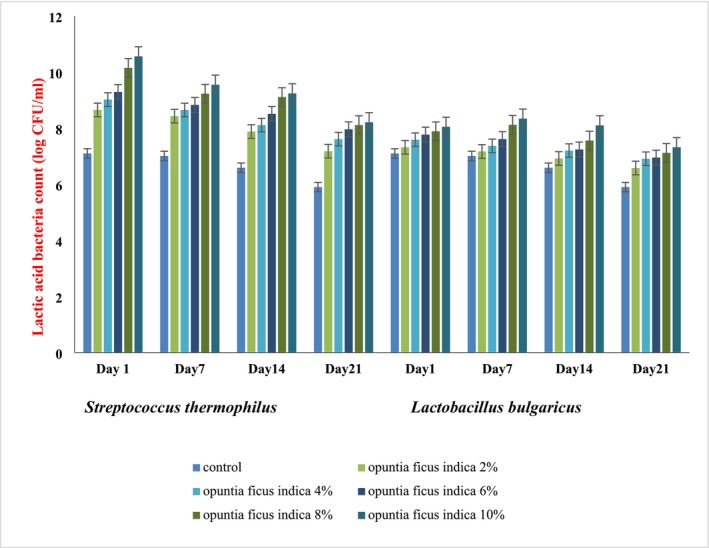
Lactic acid bacteria (LAB) viable count ( *Streptococcus thermophilus* and *Lactobacillus bulgaricus* ) in control and CPP fortified yogurt at different storage periods.

The impact of CPP inclusion at various concentrations (2%, 4%, 6%, 8%, and 10%) on the viability of LAB ( *Streptococcus thermophilus* and *Lactobacillus delbrueckii* subsp. *bulgaricus*) of yogurt was evaluated during refrigerated storage of 21 days (Figure 1). The LAB were expressed as log CFU/mL. The initial count of *S. thermophilus* in the control sample was 7.1 log CFU/mL on the first day of the storage period, which was significantly (*p* < 0.05) lower than the counts observed in all CPP‐containing yogurt samples. LAB count showed a dose‐dependent increase, with the highest count (10.56 log CFU/mL) recorded in the yogurt samples having 10% CPP. This enhanced count might be associated with the presence of fermentable sugars, polyphenols, and dietary fibers in the CPP composition that may act as prebiotic and enhance starter culture metabolic activity. A gradual declining trend was observed in *S. thermophilus* count in all yogurt samples over the storage period. However, the reduction was more pronounced in the control sample, which declined to 5.9 log CFU/mL by day 21. In contrast, the yogurt containing 10% CPP sustained a viable count of 8.5 log CFU/mL. These results demonstrated that the inclusion of CPP in yogurt samples not only increased the initial LAB count but also improved bacterial stability during storage; the reason might be attributed to the CPP antioxidative properties that reduce oxidative stress on the bacteria. *L. bulgaricus* count also follows a similar trend as *S. thermophilus* count, where the control samples exhibited the lowest count (7.10 log CFU/mL) initially on Day 1, while the 10% CPP yogurt samples recorded a significantly higher count of 8.05 log CFU/mL. By Day 21, the control declined to 6.3 log CFU/mL, whereas the 10% CPP added treatment retained 7.8 log CFU/mL. The ability of CPP to maintain a healthy viable bacterial count during storage confirms cactus pear prebiotic ability and the availability of polyphenols and fiber in its composition. Dietary fiber works as a fermentable substrate that promotes the growth of LAB and offers additional sources of carbs. Xu et al. (2022) noticed a similar pattern and discovered that adding hempseed protein to soy yogurt enhanced the amount of LAB and improved the physicochemical characteristics of the yogurt. Additionally, it has been observed that adding fruits and vegetables to yogurt increased the number of healthy bacteria (probiotic and prebiotic) and provided additional health advantages (Goktas et al. 2023; Kamber and Harmankaya 2019; Barakat and Hassan 2017). Wajs et al. (2023) examined the impacts of different pulp types made from Graviola and demonstrated that the microbiota of base yogurt remained unaffected, but the yogurt supplemented with fruit pulp increased the proliferation and viability of LAB (Wajs et al. 2023). Furthermore, the almost sustained bacterial populations throughout storage, especially in yogurt samples having 6%–10% CPP, demonstrated that cactus pear (*O. ficus indica*) pulp can improve the shelf stability and probiotic potential of yogurt. This suggests its health‐promising application in the development and formulation of value‐added functional dairy products.

### 3.6 Sensory Characteristics of Fortified Yogurt

**FIGURE 2:**
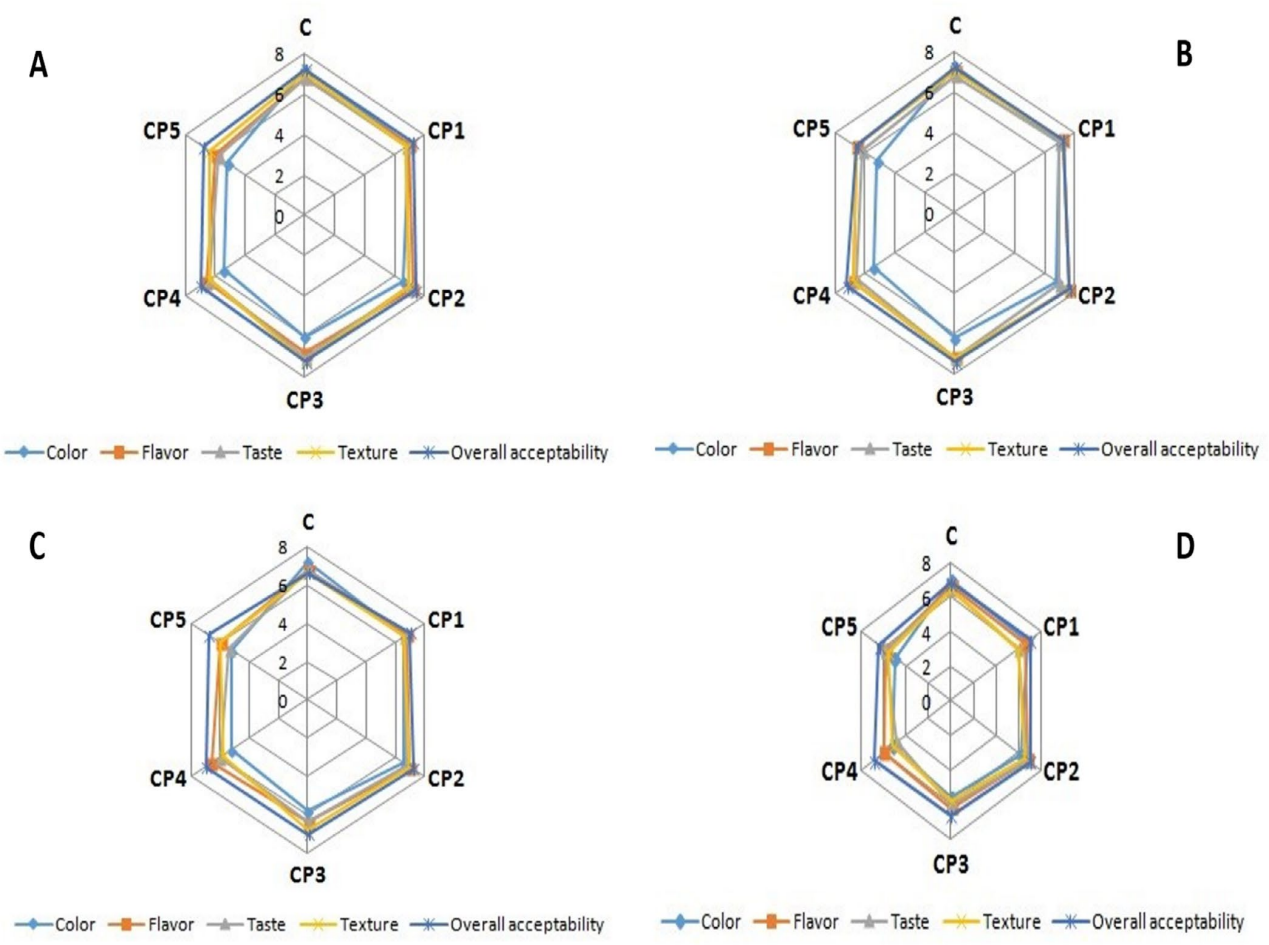
Sensory properties of yogurt at 1 day (A), 7 days (B), 14 days (C), and 21 days (D).

Sensory assessment enables the recording of human preferences in the form of ratings for numerous sensory qualities. Sensory evaluation was conducted on control and CPP (2%, 4%, 6%, 8%, and 10%) supplemented yogurt samples. Figure 2A–D shows the effects of CPP on the color, flavor, taste, texture, and overall acceptability of yogurt samples over various storage days. The color of fermented dairy products is a key factor in both production and marketing, influencing how consumers perceive and prefer the product. Control sample color values ranged from 6.2 to 7.2 across all storage periods. The color values of 2% CPP‐supplemented samples ranged from 6.2% to 7.0% over 121 days, while those of 4% CPP‐supplemented samples ranged from 6.1% to 7.00%. The color value of yogurt samples supplemented with 6% CPP remained constant at a value of 6% throughout storage. On the other hand, the color values of yogurt samples supplemented with 8% CPP ranged from 5.2% to 5.8%, whereas those supplemented with 10% CPP remained at 5.2%. Overall, the results indicated that, in contrast to the control, yogurt samples with 2% addition levels of CPP had slightly higher color values during storage; however, when addition levels were gradually increased, the color values of the supplemented yogurt samples gradually decreased. As demonstrated in Figure 2, the flavor of control samples ranged from 6.3 to 7.1 across all storage periods. The flavor of 2% CPP‐supplemented samples stored for 1–21 days ranged between 6.3 and 7.0. In the case of 4% CPP‐supplemented yogurt samples, they maintained a taste range of 6.5–7.0. The flavor of 6%, 8%, and 10% CPP‐supplemented yogurt samples ranged from 6.5 to 7.0, 6.2 to 6.9, and 6.2 to 6, respectively. In terms of flavor, a 2% addition level resulted in a minor enhancement in the yogurt flavor when compared to the control; however, higher levels of supplementation up to 4% addition resulted in comparable flavor values to the control. The 6% inclusion level did not significantly affect yogurt flavor scores (*p* > 0.05), but they did vary slightly in a decreasing manner. As depicted in Figure 2, the texture of control samples remained consistent, ranging from 6.5 to 7.0 throughout the entire storage period. Similarly, the texture of 2% CPP‐supplemented samples remained at a score of 6.5. For 4% CPP‐supplemented yogurt samples, texture ratings ranged between 6.8 and 7.0 during the 1 to 21‐day storage period. The texture of 6%, 8%, and 10% CPP‐supplemented yogurt samples varied between 6.6% and 7.1%, 6.3% and 5.9%, and 6.1% and 6.0%, respectively. In general, the inclusion of 6% produced higher texture scores than both the control group and other supplemented samples. In contrast, concentrations of 2%, 4%, 8%, and 10% caused texture scores to decrease in a concentration‐dependent manner across all storage durations. This shows that the 6% inclusion level was ideal for preserving the enhanced yogurt's sensory qualities. The taste of control samples remained between 6.4 and 7.0. The taste of samples supplemented with 2% CPP for 1–21 days ranged between 6.4 and 6.5. The flavor of 4% CPP‐supplemented yogurt remained consistent at 6.7–7.1 over storage. The taste of 6%, 8%, and 10% CPP‐supplemented yogurt samples varied from 6.6 to 6.8, 6.2 to 6.0, and 5.9 to 5.2, respectively. Regarding flavor scores, the addition levels of 2%, 6%, and 8% showed no notable impact on the taste of supplemented yogurts, with panelists consistently assigned similar scores; however, the 10% addition levels caused minor declines in the taste of yogurt samples across all storage durations.

For the control sample, acceptability scores ranged from 7.1 to 6.7 during storage durations of 1–21 days. Overall acceptability scores for 2% of CPP‐supplemented samples were constant (7). Overall acceptance scores for 4%, 6%, and 8% of CPP‐supplemented yogurt samples ranged from 7.0–7.1 to 7–7.1. The total acceptance scores for 10% CPP‐supplemented yogurt samples were constant (7) throughout the storage period. Overall, scores for all sensory qualities increased with storage periods. The results demonstrated that all items, including the control, had similar overall acceptability scores as storage time varies from 1 to 21 days. Similarly, Salih et al. (2020) examined the sensory qualities of yogurt prepared from cow milk after adding gum arabic powder at concentrations ranging from 0.5% to 1.5%. The scientists found that adding 0.5% gum arabic powder to yogurt had a substantial (*p* < 0.05) effect on its sensory qualities, with the greatest overall acceptability score.

Incorporating CPP at moderate concentrations (4%, 6%, and 8%) improves sensory qualities. However, adding higher concentrations (10%) of pulp harmed the sensory qualities. Furthermore, the length of the storage time had a negative impact on the sensory scores. The yogurt sample with 4% pulp had the highest sensory scores, whereas the sample with 10% pulp had the lowest. Previous research found that adding different fruits improved the sensory qualities of yogurt (Kamber and Harmankaya 2019; Farag et al. 2022). Other scholars (Xu et al. 2022; Grasso et al. 2020; Bulut et al. 2022; Fan et al. 2022) have determined that the addition of plant‐based additives increases the sensory scores of yogurt samples. The enhanced sensory ratings can be linked to the favorable color and flavor of CPP, which enhance the sensory attributes of yogurt. The quality of yogurt is determined by the source and kind of milk, the variety of cactus pear, variations in milk composition, and cactus pear grown under various climatic, environmental, and seasonal conditions.

## 4 Conclusion

This study concludes that the incorporation of cactus pear pulp (CPP) in buffalo milk yogurt significantly improved its quality characteristics, including physicochemical, functional, microbial, and sensory attributes in a concentration‐dependent manner. The inclusion of CPP significantly reduced the syneresis susceptibility and improved the water‐holding capacity, particularly at higher concentration levels (8% and 10% inclusion levels). In samples with 10% CPP, significantly higher total phenolic content and DPPH radical scavenging activity were noticed, confirming the functional antioxidant potential of the formulation. Furthermore, the good and constant LAB viability count during the early storage period in CPP‐enriched yogurt samples reflects a favorable environment for probiotic activity supported by the prebiotic effect of the CPP inherent bioactive compounds. Inclusion of CPP (6%–10%) seems to be the optimal level for maintaining viable bacterial populations above the critical level of 10^6^ CFU/mL, necessary for presenting health benefits throughout the storage period. Moreover, sensory evaluation demonstrated that yogurt samples (4% CPP) received the highest overall acceptability scores as achieving the best balance for taste, texture, and appearance. This suggests that moderate CPP addition levels may optimize consumer appeal while still delivering functional benefits. In conclusion, CPP may be utilized in the production of yogurt with improved quality and therapeutic properties. Moreover, it is suggested that CPP can be utilized in the development of other innovative, health‐promoting dairy products.

## Author Contributions

**Farzana Siddique:** Conceptualization, Supervision (equal), Writing ‐ review and editing (equal). **Muhammad Arshad:** Data curation. Project administration (equal). **Rashid Ahmad Khan:** Formal analysis, Methodology, Visualization (equal). **Muhammad Qasim Ali:** Investigation (equal), Resources (equal), Validation (equal). **Sidrah:** Investigation (equal), Software (equal), Validation (equal). **Muhammad Siddique Raza:** Software (equal), Roles/Writing ‐ original draft (equal). **Muhammad Naeem Zubairi:** Resources (equal), Roles/Writing ‐ original draft (equal). **Nida Firdous:** Supervision (equal), Visualization (equal), Writing ‐ review and editing (equal). **Ashiq Hussain:** Roles/Writing ‐ original draft (equal), Writing ‐ review and editing (equal). **Henock Woldemichael Woldemariam:** Funding acquisition, Project administration (equal).

## Ethics Statement

The sensory evaluation was conducted in accordance with the guidelines for sensory studies as outlined by the Ethics committee of Institute of Food Science and Human Nutrition, University of Sargodha, with the approval number (UOS/IFSN/2024/06).

## Consent

Informed consent was obtained from all individual participants included in the study.

## Conflicts of Interest

The authors declare no conflicts of interest.

## Data Availability

All data generated or analyzed during this study are included in this published article.
